# Supplementary material for: Combinatorial Inhibition of Cell Surface Receptors Using Dual Aptamer-Functionalized Nanoconstructs for Cancer Treatment
Source: Pharmaceutics. 2020 Jul 21;12(7):689. doi: 10.3390/pharmaceutics12070689 (PMC7408393; doi:10.3390/pharmaceutics12070689)
Supplement: Supplementary file 1 [file pharmaceutics-12-00689-s001.pdf]

pharmaceutics

Supplementary Materials: Combinatorial Inhibition of Cell Surface Receptors using Dual Aptamer-functionalized Nanoconstructs for Cancer Treatment

Hyojin Lee , Tae Hee Kim, Daechan Park, Mihue Jang, Justin J. Chung, Soo Hyun Kim, Sang-Heon Kim, Kwan Hyi Lee, Youngmee Jung and Seung Ja Oh

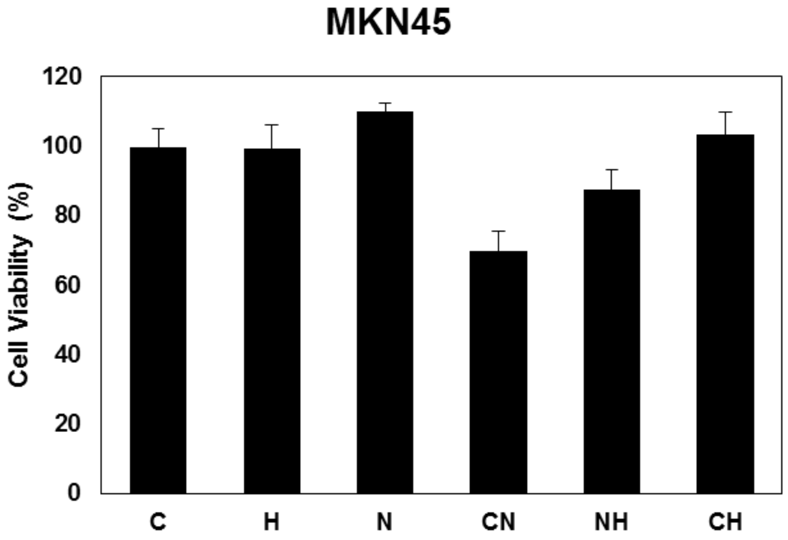

Figure S1. The result of cell viability assay. MKN-45 cells were treated with 250 nM single aptamer-AuNS and bi-functional AuNS with different combinations of aptamers. The viability of MKN-45 cells dramatically decreased in AuNS-CN condition as compared with AuNS-NH, AuNS-CH, and single-aptamer-AuNS.

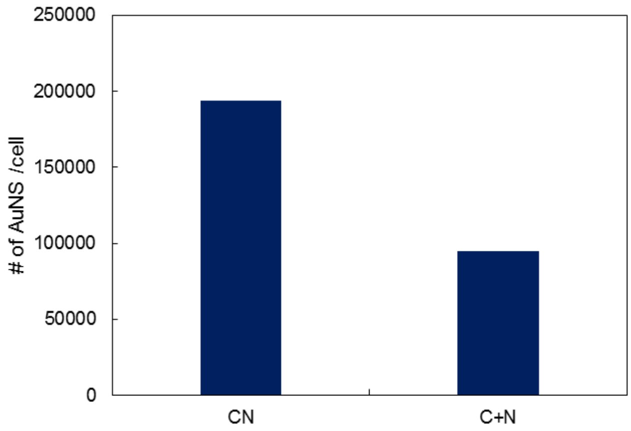

Figure S2. The result of ICP-MS analysis. After incubation of MKN-45 cells with 250 nM aptamer-AuNS, the amount of Au internalized was analyzed using ICP-MS. The result for ICP-MS suggest that bi-functional AuNS were effectively delivered into cells as compared with a mixture of anti-nucleolin-AuNS and anti-c-Met-AuNS.

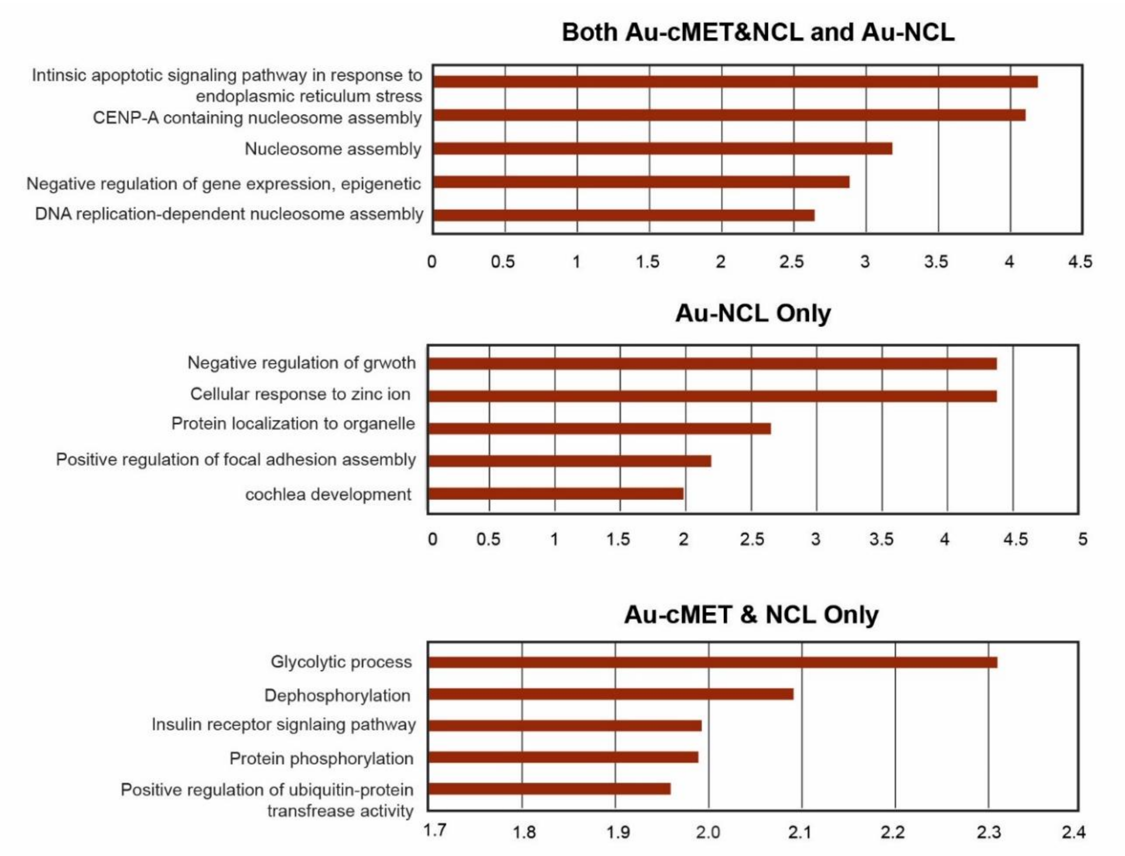

Figure S3. Gene ontology analysis for overlapped or unique differentially expressed genes between nucleolin-AuNS and c-Met-nucleolin-AuNS treatment. The top GO term for shared DEGs between nucleolin-AuNS and c-Met-nucleolin-AuNS treatment was apoptosis-related, whereas the unique term with c-Met-nucleolin-AuNS treatment was phosphorylation-associated signaling pathway. X-axis represents −log10 (p-value).

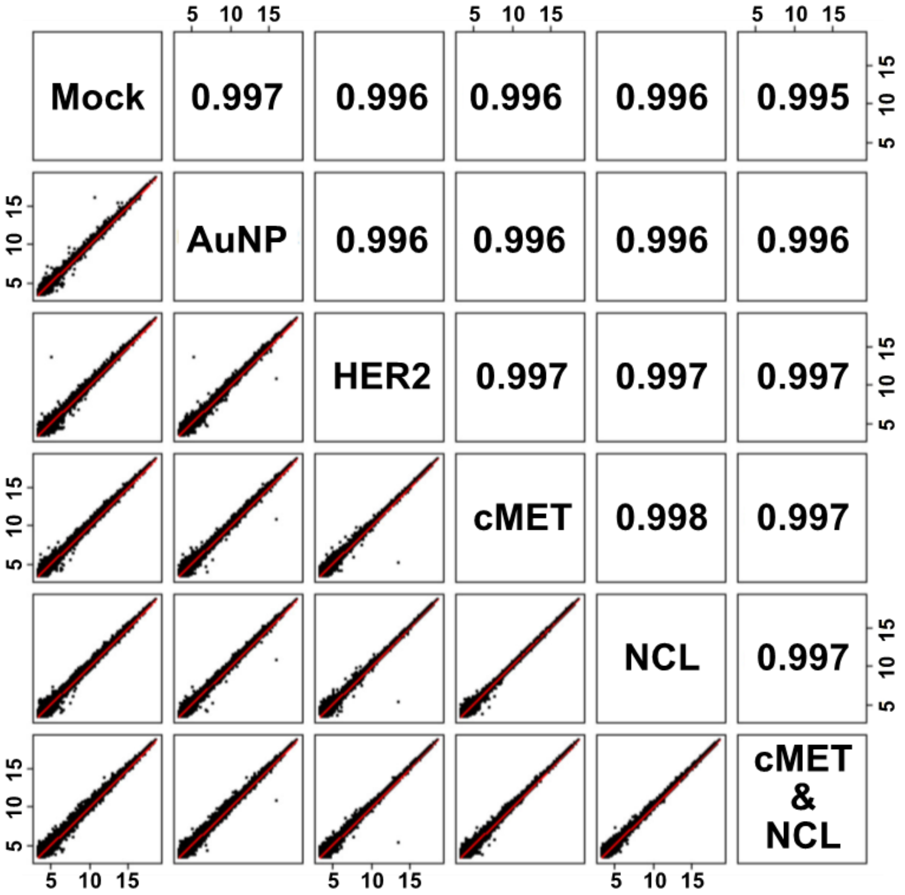

Figure S4. Pair wise comparison of gene expression profiles. log2 probe intensities were compared in pair wise manner, and the Pearson correlation coefficients were calculated. Overall transcription profiles were stable upon the treatments.
